# Acquired Vertical Accommodative Vergence

**DOI:** 10.2174/1874364100802010034

**Published:** 2008-03-08

**Authors:** Ulrike Klein-Scharff, Guntram Kommerell, Wolf A Lagrèze

**Affiliations:** Universitäts-Augenklinik, Freiburg, Germany

**Keywords:** Strabismus, vergence, vertical, accommodation.

## Abstract

Vertical accommodative vergence is an unusual synkinesis in which vertical vergence is modulated together with accommodation. It results from a supranuclear miswiring of the network normally conveying accommodative convergence. So far, it is unknown whether this condition is congenital or acquired. We identified an otherwise healthy girl who gradually developed vertical accommodative vergence between five to 13 years of age. Change of accommodation by 3 diopters induced a vertical vergence of 10 degrees. This observation proves that the miswiring responsible for vertical accommodative vergence must not necessarily be congenital, but can be acquired. The cause and the mechanism leading to vertical accommodative vergence are yet unknown.

## INTRODUCTION

In 1991, we described five patients who showed a modulation of vertical vergence position as a function of accommodation [1]. We named this synkinesis “vertical accommodative vergence” (VAV). VAV indicates a supranuclear miswiring of the network normally concerned with accommodative convergence. During the following years, a number of similar cases have been reported [2-4]. Follow-up has been short in all these cases, and spontaneous changes of VAV have not been observed. Therefore, it has been suspected that this condition might be congenital. Recently, we found VAV in a 13-year-old girl whom we had seen 10 years earlier, without VAV at that time. This observation challenges the concept that VAV has to be congenital.

## MATERIALS AND METHODOLOGY

The case of a young female patient is presented. She and her mother gave her informed consent to this publication which adheres to the 1964 declaration of Helsinki.

## RESULTS

At the age of 13 years the above mentioned patient complained of blurred vision at near and occasional double vision, particularly when reading. To overcome this problem she enlarged the reading distance.

**Fig. (1).**
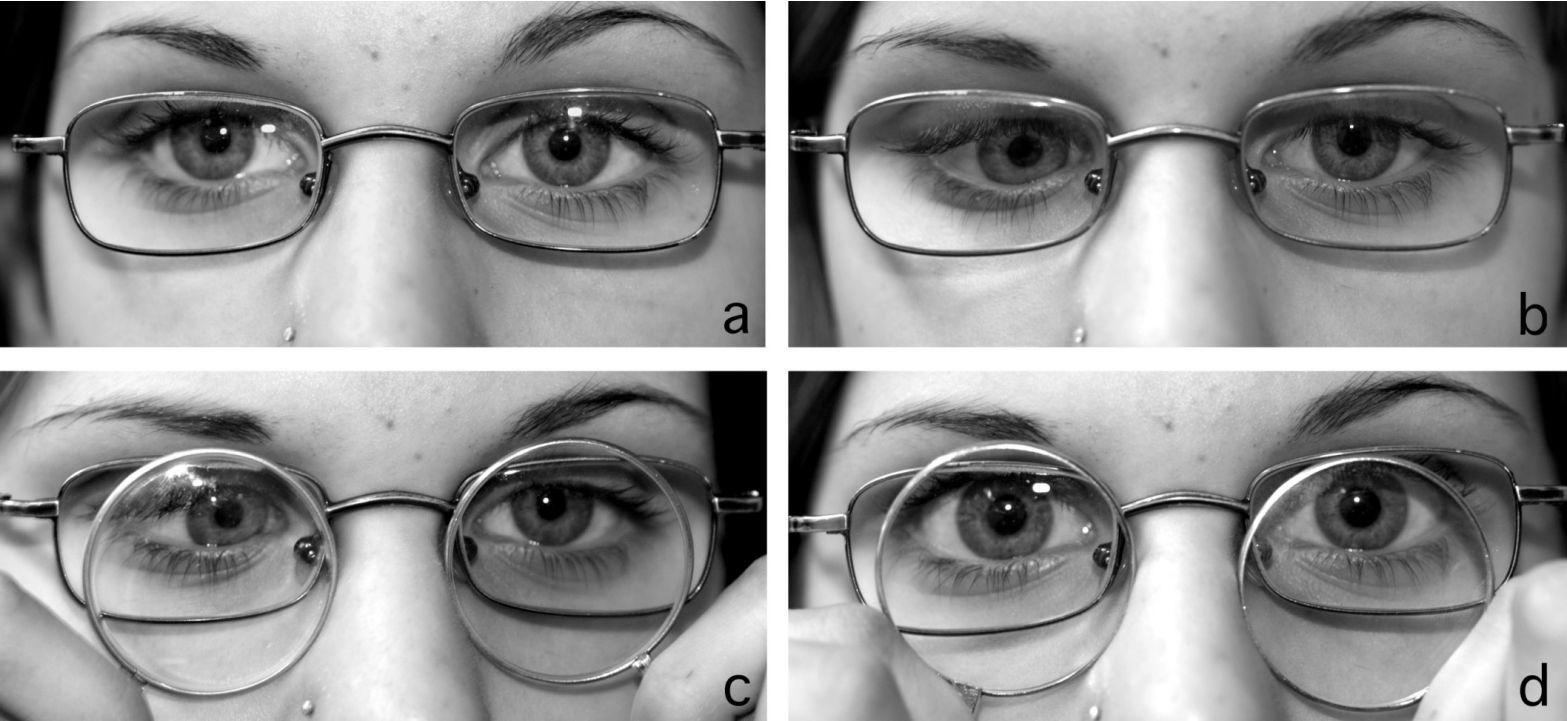
Patient with acquired vertical accommodative vergence. **(a)** fixating at distance and refracted for distance*, **(b)** fixating at near and refracted for distance, **(c)** fixating at distance with an addition of -3 D to distance refraction, and **(d)** fixating at near with an addition of +3 D to distance refraction. *(a) may indicate right hypotropia and exotropia. However, the corneal light reflex has the same height in both eyes in relation to the upper pupillary margin. This may be difficult to see as the upper part of the right iris appears darker than in left eye. With regard to a possible horizontal deviation one has to keep in mind that the patient was fixating at distance while being photographed from 1m explaining the nasal displacement of the light reflex.

Cover testing was performed with the eyes refracted for distance (both eyes +5.5 D). At distance (5 m), there was a right hypophoria of 3° (no manifest deviation, Fig. **1a**). At near (30 cm), the hypophoria increased to 14° with an additional esophoria of 10° (Fig. **1b**). Occasionally and only at near the phoria decompensated into a manifest strabismus, without double vision in most instances. Minus 3 D increased the deviation at distance from +3° L/R 3° to +16° L/R 18° (Fig. **1c**), corresponding to a vertical vergence movement of 15°. An addition of +3 D reduced the right hypophoria at near from L/R 14° to L/R 4° (Fig. **1d**). This means that a change of accommodation by 3 D induced a vertical vergence movement of 10^°^.

Measuring of the angle in different directions of gaze by turning and pitching the head was done at the Harms tangent screen in 2.5 m with a dark red glass before the left eye. The results showed a hypotropia of the right eye which increased from 3° in straight gaze to 8° in 20° adduction. There was no difference of the vertical or horizontal angle between up- and down gaze. The deviation did not change on head tilt to the left or right. Additionally, we measured the angle in the four tertiary positions, using the alternate prism cover test with the left eye fixating 25° away from the mid position. As fixation targets we used accommodation-demanding letters attached on two tangent screens, one at 2.5 m and another at 0.33 m. The angle was about the same in the four tertiary positions, as was the increase of the angle at near. There was an excyclotropia of 8°, measured with a Maddox cylinder in front of the left eye, both at 2,5 m and at 0,33 m. A dissociated vertical deviation (DVD) was excluded with the reversed fixation test [5]. Monocular goal-directed saccades and optokinetic responses were normal, both in the horizontal and vertical direction. Stereo resolution down to 40 arcsec was shown with the Titmus Stereo Test. The motility of the lids was normal. The pupils reacted normally to light and near. Refracted for distance, the near point of accommodation was 13 cm in both eyes. The cycloplegic refraction measured was +5,5 D in the right eye and +5.5 D in the left eye. The visual acuity under refraction was 1.25 (7.5/6) in each eye. To improve sight at near, we offered plus-lenses for prolonged reading, but the patient told us that she could cope without them.

### Previous Findings

The girl had been referred to us for the first time at the age of three because her eyes had become esotropic two weeks previously. Spectacle correction of a hypermetropia and muscle surgery restored binocularity for distance. For near, esotropia prevailed at first but was occasionally overcome by fusion during the following years. First signs of VAV appeared at the age of five, and became more pronounced on subsequent examinations. Details of the previous findings, all of them documented by the first author, are summarised in Table **1**.

## DISCUSSION

The 13-year-old girl showed the characteristic signs of vertical accommodative vergence (VAV): looking from 5 m to 30 cm led to a vertical vergence of 11°, relaxation of accommodation with an addition of +3 D at near induced a vertical vergence of 10°, and stimulation of accommodation with -3 D at distance induced a vertical vergence of 15°.

### Differential Diagnosis

A dissociated vertical divergence was excluded with the reversed fixation test [5]. The further depression of the right eye from straight gaze to adduction was only 5°, i.e. too small to explain the vertical vergence as a consequence of near convergence. An aberrant regeneration of the oculomotor nerve was ruled out as the cause of the synkinesis between accommodation and vertical vergence, because the motility of the pupils and the upper lids was normal. Despite an excyclotorsion of 8°, a fourth nerve palsy is unlikely, because the vertical deviation decreased in right gaze and did not change between up- and down-gaze.

### Time Course

During the first three years of live the eyes were unconspicuous. At the age of three years an esotropia with elevation in adduction of the left eye appeared. At this age and at the age of four (after eye muscle surgery), the esotropia increased when the girl looked at near, but there was no evidence of a vertical vergence. It is unlikely that the examiner (first author) overlooked VAV at this time, for two reasons. First, she measured, not merely estimated, the angle at far and near. Second, she was well aware of VAV, since she had introduced this condition into the literature, just three years before she saw the three year-old girl [1]. These findings exclude that the synkinesis between accommodation and vertical vergence was congenital. A first sign of VAV appeared not before the age of five, when the girl showed a vertical angle, only at near (L/R 6°). Until the age of 13 years, the VAV gradually increased up to an angle of about 13°. Hence, the VAV must have developed between the ages of five and 13 years.

### Cases in the Literature Suggesting Acquisition of VAV

Although most cases of VAV reported in the literature are compatible with a congenital synkinesis, there are three patients in whom an acquisition of VAV later in life appears to be likely. One of the patients described in our previous publication [1] complained of vertical diplopia at near that did not occur before the age of about 16 years. Gräf [2] described a 31 year-old man with VAV whose right eye had become blind from an injury at the age of nine. The history and a photograph taken at the age of five suggested that there had been no strabismus before the injury. Thomas *et al*. [4] reported a 36 year-old patient with VAV who had been complaining of worsening intermittent double vision only for the last eight years.

### Pathogenesis

We have no compelling suggestion as to the mechanism that leads to VAV. A general disease has not been implicated in any of the cases reported in the literature. Similar to the girl described in the present paper most patients had binocular vision, broken up only occasionally by manifest strabismus. In none of them did VAV facilitate binocularity. Therefore, an advantageous adaptation to an underlying ocular motor abnormality can be ruled out. Rather, VAV appears to be a primary dysfunction of the supranuclear ocular motor system.

We have considered that VAV may be interpreted as an exaggeration of the normal cross coupling between the near triad and the vertical vergence that occurs when a target is pursued from far to near in a tertiary direction of gaze. For example, when the target is moved in the upper right field of gaze along the left eye’s line of sight from distance to proximity, binocular fusion can be maintained only if the right eye rises in relation to the left eye, because the right eye is nearer to the target than the left eye. This type of cross coupling between the near triad and the vertical vergence is preprogrammed in the ocular motor system, enabling sustainance of binocularity even during rapid eye movements [6]. However, as we did not find an accentuation of VAV in any of the tertiary directions of gaze, we doubt that this physiological cross coupling is related to VAV.

## CONCLUSION

VAV is a synkinesis between accommodation and vertical vergence, indicating a supranuclear miswiring of the network normally concerned with accommodative convergence. This miswiring may occur as a congenital abnormality, but can be acquired without indicating a general disease. The cause and the pathogenesis of VAV are unknown.

## Figures and Tables

**Table 1.** Summary of Previous Findings

Age [y]	Refraction [D]	Angle F [°]	Angle N [°]	Change of VD with Horizontal Gaze	Binocularity	Intervention
3	+ 3.25 OD + 3.50 OS	+ 11	+ 22	Elevation in adduction OS	Constant esotropia	Recession both medial recti 7 mm, recession left inferior oblique 6 mm
3		- 2 R/L 2	+ 2 R/L 1	Slight elevation in adduction OD		
3		0 R/L 2	+10 R/L 3	No comment in the chart	F: compensated N: occasionally compensated	Bifocals prescribed, not tolerated
4	+ 4.50 OD + 4.50 OS	+ 4 R/L 0	+ 18 R/L 0	No comment in the chart	F: compensated N: occasionally compensated	
5	+ 5.50 OD + 5.50 OS	0 R/L 0	+ 7 L/R 6	Elevation in adduction OS	F: compensated N: never compensated	
11		+4 L/R 3	+ 10 L/R 12	Depression in adduction OD, no A pattern	F: compensated N: mostly compensated	

The angles at far (F = 5 m) and at near (N = 30 cm) were measured with the alternate prism cover test while the eyes were refracted for distance. “Compensated” means that there was no manifest deviation in the unilateral cover test. VD = vertical deviation, OD = right eye, OS = left eye, + = esodeviation, - = exodeviation, R/L = right over left eye, L/R = left over right eye.
